# An Ambient Measurement Technique for Vehicle Emission Quantification and Concentration Source Apportionment

**DOI:** 10.1021/acs.est.4c07907

**Published:** 2024-11-04

**Authors:** Naomi J. Farren, Samuel Wilson, Yoann Bernard, Marvin D. Shaw, Kaylin Lee, Mallery Crowe, David C. Carslaw

**Affiliations:** † Wolfson Atmospheric Chemistry Laboratories, University of York, York YO10 5DD, United Kingdom; ‡ The International Council on Clean Transportation, Fasanenstr. 85, Berlin 10623, Germany; § National Centre for Atmospheric Science, University of York, York YO10 5DD, United Kingdom

**Keywords:** vehicle emissions, plume sampling, regression, source apportionment, ammonia

## Abstract

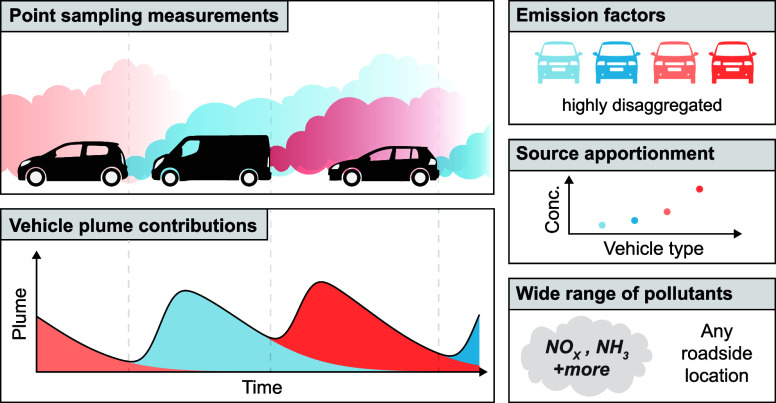

We develop a new technique called *plume regression* where fast response instruments located at the roadside are used to measure exhaust plumes of passing vehicles. The approach is used to generate highly disaggregated vehicle emissions information by vehicle type, which compares well with traditional vehicle emission remote sensing. Additionally, the technique provides valuable new information on ambient concentration source apportionment by vehicle type. The technique is flexible enough to consider a wide range of air pollutants and be deployed at roadside ambient monitoring locations. The new approach is used to quantify emissions and concentration source apportionment for ammonia (NH_3_) and nitrogen oxides (NO_*x*_). We find that emissions of NH_3_ are generally very well controlled from diesel vehicles including those with selective catalytic reduction systems that use NH_3_ to reduce emissions of NO_*x*_. By contrast, gasoline passenger cars are shown to be the dominant contributor to NH_3_ emissions, which increase with vehicle mileage. Average fuel-specific NH_3_ emission factors for gasoline vehicles range from 0.3 to 1.2 g kg^–1^, while diesel vehicle emission factors remain below 0.06 g kg^–1^, with the exception of Euro VI buses with the latest regulatory provisions (0.5 g kg^–1^).

## Abstract 2

Emission control technologies have led to reduced but increasingly complex emissions from the transport sector. We present a new technique to characterize these emissions and apportion roadside pollutant concentrations to specific vehicle types.

## Introduction

The implementation of policy measures and emission control technologies in the UK and EU-27 countries in recent decades has led to a reduction in emissions of many high priority pollutants from the transport sector, including nitrogen oxides (NO_*x*_), sulfur oxides, carbon monoxide, and particulate matter (PM).^1^ However, road transport emissions of pollutants such as ammonia (NH_3_) and nitrous oxide (N_2_O) have recently increased, due to their formation as unintended byproducts of aftertreatment technologies designed to minimize NO_*x*_, including three way catalysts (TWCs) and selective catalytic reduction (SCR) systems.^2,3^ NH_3_ contributes to the formation of harmful fine airborne particles in the atmosphere and excess nitrogen deposition in ecosystems,^4,5^ while N_2_O is a long-lived greenhouse gas and ozone-depleting substance.^6,7^ Another challenge for the transport sector is nonexhaust PM, arising from sources such as brake wear and tire abrasion. Due to increasingly widespread adoption of aftertreatment systems and increased electrification of the transport sector, these pollutants are expected to persist as the fleet modernizes. As emissions legislation becomes increasingly stringent, it is critical to develop new measurement techniques and data processing methods for accurate emissions quantification.

Traditionally, remote sensing (RS) has been used as an unobtrusive technique for measuring vehicle exhaust emissions during normal, on-road operation.^8,9^ RS measurements have been conducted globally and the data provides real-world evidence for the efficacy of emission control technologies and vehicle emission policies, evaluating the impact of specific driving conditions, and performing fleet surveillance studies.^10^ The instrumentation is deployed across a single lane of traffic and individual vehicle exhaust plumes are measured over a short duration of about 0.5 s. A single vehicle RS measurement represents instantaneous emissions and carries high uncertainties as to how the vehicle performs. Therefore, multiple measurements of a single vehicle type, e.g., a Euro 5 passenger car, must be aggregated to generate an emission estimate with an associated uncertainty. Tailpipe pollutant emissions are measured as ratios to CO_2_, from which fuel-based emission factors (EFs) can be derived using carbon-balanced assumptions related to the combustion of the fuel.^11^ The main limitation of RS is the suite of pollutants that can be measured. There is limited scope to measure gaseous species of increasing concern, such as NH_3_, N_2_O and speciated hydrocarbons. RS systems lack accuracy for particulates; they are not sensitive enough to quantify PM from modern vehicles and do not measure metrics such as particle number (PN) or black carbon (BC).^12^

An alternative approach to RS is *point sampling* (PS), where continuous fast response measurements are made at the curbside and the plumes from individual vehicles are measured.^13−15^ By simultaneously measuring CO_2_ and pollutants such as NO_*x*_ and integrating the concentrations, emission ratios determined. In principle, any air quality instrument with sufficient response time and accuracy can be deployed, which drastically broadens the suite of gaseous pollutants and particle metrics that can be measured compared to RS. Nevertheless there are several important practical constraints which currently affect PS. The principal constraint is having enough of a time gap between vehicles to isolate an individual vehicle plume from potential interference from other nearby vehicles. A “Goldilocks” location is preferred, that is not too busy (to avoid dispersing plumes overlapping) but busy enough to make the effort of measurement worthwhile—in practice, selecting such a location is challenging. In Knoll et al. for example, approximately 70% of the data is disregarded due to potential plume interference.^11^ There are also other potential issues such as the effect of meteorology, whereby a plume may not be detected at all due to an unfavorable wind direction.

In this paper, we develop a new way in which PS data can be analyzed to provide vehicle-specific EFs. Rather than seek to identify and quantify individual vehicle plumes of CO_2_ and the measured pollutants, we consider the problem from the perspective of the likelihood of observing a plume based on an average plume profile. The approach, termed *plume regression*, is efficient and practical and uses data from all vehicle passes. In addition to providing highly disaggregated vehicle emissions information by vehicle type, this new approach provides valuable information on ambient concentration source apportionment. Here we deploy highly accurate, portable and fast response NH_3_, NO_*x*_ and CO_2_ analyzers at the curbside and use the new plume regression technique to quantify emissions *and* concentration source apportionment for vehicular NH_3_ and NO_*x*_.

## Materials and Methods

### Instrumentation

Fast response air quality instrumentation was deployed on the curbside to perform continuous measurements of vehicle plumes from passing vehicles. High resolution measurements of NH_3_, NO_*x*_ (NO and NO_2_) and CO_2_ were made. The NH_3_ analyzer (model HT8700E) is a quantum-cascade laser (QCL) based system which uses wavelength modulation spectroscopy to determine in situ concentrations of NH_3_ at 10 Hz resolution.^16^ The QCL emits a continuous beam at 9.06 μm into an open-air Herriott cell with concave mirrors at each end. The open-path design avoids challenges associated with surface adsorption of NH_3_. Further technical details are described in Wang et al.^17^ and the instrument calibration procedure is described in the Supporting Information.

NO_*x*_ and CO_2_ were measured using an Iterative Cavity enhanced Differential Optical Absorption Spectrometer (ICAD) developed by Airyx.^18^ The ICAD uses optical absorption spectroscopy in the spectral range between 430 and 465 nm to directly measure NO_2_, with a t90 time of 1 s. An internal converter based on gas phase titration with O_3_ converts NO to NO_2_, allowing for total NO_*x*_ and NO concentrations to be derived. Atmospheric CO_2_ concentrations are measured simultaneously using an in-line nondispersive infrared gas sensor. Additional technical information is described in Horbanski et al.^19^

A custom-built vehicle measurement device was deployed adjacent to the air quality instrumentation to record vehicle pass times. The device uses optical sensors to calculate vehicle speed and acceleration, and trigger the camera to capture an image of the rear of each passing vehicle. Automatic License Plate Recognition software (Rekor CarCheck Plus) is used to extract the license plate data from these images.^20^

### Measurement Surveys

PS surveys were conducted at three locations in the city of York, UK. Two of the measurement sites were based on the University of York campus (53.947, −1.047) and the third site was near a large retail park in an industrial area (Clifton Moor Gate, 53.987, −1.103). The surveys were carried out on weekdays between September and November 2023 during daylight hours and periods of dry weather. Ambient temperatures ranged from 1.9 to 18.7 °C, with a mean temperature of 12.9 °C. The HT8700E and ICAD were placed on a platform trolley at the curbside. The HT8700E open-air cell and the ICAD sample line (30 cm length of 1/4″ diameter PFA tubing) were positioned next to each other, close to the diluting exhaust plumes from passing traffic. The vehicle measurement device was placed approximately 1 m upstream of the analyzers and the vehicle registration plate camera was a further 5–10 m upstream. All equipment was powered using two Anker portable power stations with 512 Wh and 256 Wh capacity. Photographs of the measurement sites are shown in Figures S1–S3.

The license plate data was sent to CDL Vehicle Information Services Limited to obtain individual vehicle technical information. CDL is a commercial supplier and the data is sourced from data collected as part of the UK vehicle taxation system (DVLA) and data queried from the Society of Motor Manufacturers and Traders (SMMT) Motor Vehicle Registration Information System. A vast range of information is provided, including vehicle engine size, fuel type, make and model, date of registration, and the emission standard. The mileage of a vehicle at its last annual test of vehicle safety and roadworthiness (MOT test) is provided for vehicles over three years old.

A total of 11,264 vehicle passes were recorded over 9 measurement days and 95% of the vehicle registration plates were assigned vehicle technical information. The mean speed and acceleration of passing vehicles was higher at Clifton Moor Gate compared to the University campus (44.2 km h^–1^ and 4.8 km h^–1^ s^–1^ vs 34.5 km h^–1^ and 0.9 km h^–1^ s^–1^ respectively). A summary of the measured vehicle fleet composition and driving conditions is provided in Table S1.

### Plume Regression

**Figure 1:**
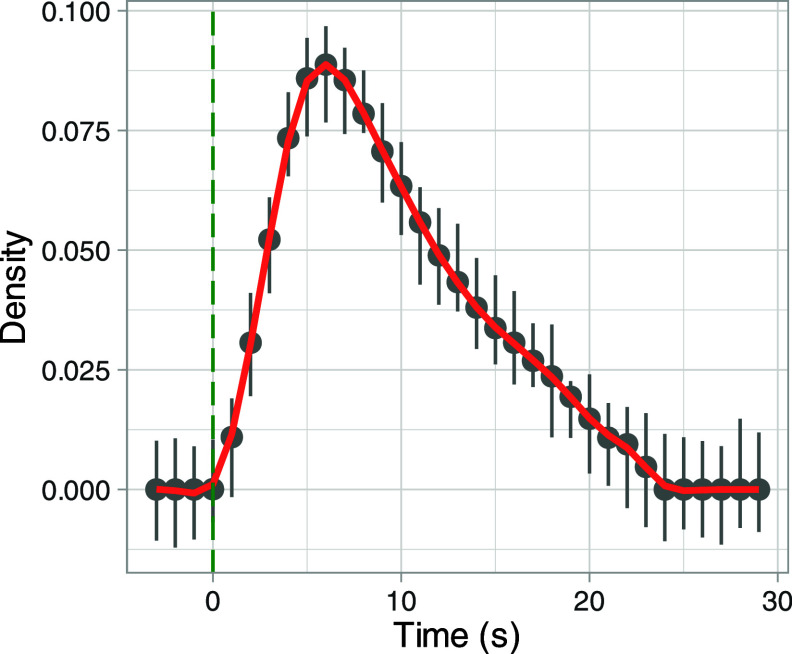
Mean plume profile compiled using CO_2_ plumes derived from vehicle passes with at least a 20 s interval before and after other vehicle passes with 95% confidence intervals shown. Time on the *x*-axis is the time since the front of a vehicle is detected. The plume has been normalized so that the area under it equals 1.

The approach starts with identifying when a vehicle passes the instrument(s) and considers the mean plume shape of CO_2_. To reduce the potential for overlapping plumes, the mean plume shape is derived from data associated with vehicle passes that have a 20 s time gap between vehicles. Considering a 20 s time gap reduces the number of measurements available (306 vehicle passes out of 10,252 in total) but provides a clear plume profile, as shown in Figure 1. The profile shown in Figure 1 varied little by the main vehicle types and pollutants (Figure S4), where there tends to be a rapid rise in concentration followed by a slower falloff; although there is less certainty with heavy good vehicles (HGVs) where there are fewer measurements. In essence, the plume profile provides a probability that a plume will be detected from a vehicle *on average* across all sites and conditions sampled. In some situations and with larger data sets, there could be merit in using different plume profiles e.g., for trucks with vertical exhaust pipes, which were not present in this study.

**Figure 2:**
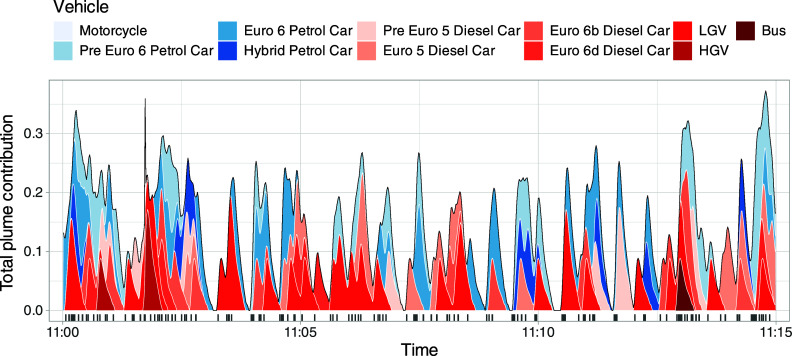
Simulated plume profiles for a typical 15 min PS measurement period, created using the average plume profile shown in Figure 1. The colors of the stacked plume profiles represent different vehicle types, chosen to illustrate both the importance of plume overlap and the analysis approach that is developed. The vertical lines on the *x*-axis show the times when a vehicle passes based on when the front of the vehicle blocks the light beam.

The mean plume profile can be used to illustrate the issue of plume overlap and interference from vehicles other than the one being measured. Taking the average plume shape in Figure 1 and adding it to the time series each time a specific type of vehicle passes generates a plot like Figure 2. A sample period of 15 min is shown where vehicles of different types pass the instruments. The choice of vehicle type grouping is only to illustrate some important issues. First, it is clear from Figure 2 that there is no single period where a vehicle plume exists in isolation and that there are always contributions from preceding vehicles. This situation is typical of PS and can only be mitigated by choosing a quieter location.

Second, Figure 2 shows that at any point in time there can be a range of contributions from different vehicles depending on both the type and proximity of a vehicle to the one being measured. The analysis approach adopted is as follows. The vehicle categories are decided ahead of the analysis e.g., by fuel type and broad vehicle category, such as those shown in Figure 2. For each category a column is added to the time series data set, which is initially set to zero. Each time one of the vehicles in the category passes the instrument, the normalized plume profile is added to the column for that particular vehicle category. This process is repeated for all vehicles that pass the instrument. Using Figure 2 as an example, 11 vehicle categories are considered. The sum of any category column is equivalent to the total number of vehicles in that category, as the area under each plume equals one.

The second step is to calculate an increment in concentration above local background for each pollutant, which represents the contribution from vehicles. There are various ways this can be approached, but a simple rolling average low percentile was chosen^21,22^ with a time width of 100 s. A width of 100 s was selected to comfortably straddle the typical width of a plume (Figure 1) but avoid capturing contributions other than a passing vehicle.

The next step is to relate the 1 Hz time series of pollutant concentration increments to the plume weighting categories from the first step to calculate the contributions from different vehicle categories that best explain the increment in concentration of the pollutant in question. There are several options for regression, but robust linear regression has been chosen to avoid the potentially influential effect of outliers(MASS).^23^ While ordinary linear regression provides mostly similar results to robust regression, outliers can invalidate the assumptions used in linear regression e.g., normally distributed residuals. There is close to zero correlation between categories of vehicles, which is beneficial from a regression perspective where multicollinearity can be problematic. Low correlation is expected because vehicles are separated in time (even if there is plume overlap) and there is effectively a random order of plume overlap by vehicle type e.g., a Euro 5 gasoline car does not always follow a Euro 5 diesel car. The inherently low correlation between vehicle groupings also means that the regression models can have many terms (tens or more) and still distinguish between them with low standard error.

The regression equations take the form:
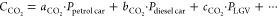
1
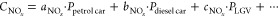
2
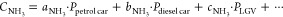
3where, ,  and  are the concentration increments for CO_2_, NO_*x*_ and NH_3_, respectively. The *P* terms represent the plume weightings for each class of vehicle considered. The *a*, *b*, and *c* terms represent the regression coefficients to be determined and represent the amount a normalized vehicle plume has to be multiplied by to give the best overall fit to the data for each vehicle category. The regression coefficients are then used to calculate other quantities such as fuel-based EFs derived from the molar ratio of NO_*x*_ or NH_3_ to CO_2_ and assumptions concerning the carbon content of fuels.

The approach above has significant benefits over approaches that attempt to isolate individual vehicle plumes. First, *all* the data and every vehicle pass is used in the regression approach, whereas typically only 10–30% of the data is used in the former approach owing to plume overlap. This aspect of the regression plume approach also greatly simplifies the analysis, as no time needs to be spent trying to identify and isolate individual vehicle plumes. The regression approach accepts that there is plume overlap but uses that information directly to determine relative contributions to concentrations by vehicle type.

There is an interesting comparison that can be made with traditional RS where typically a measurement of duration 0.5 s is made of individual exhaust plumes. The RS approach has the benefit of being vehicle-specific and providing a measurement of the plume immediately after exiting from the exhaust. However, RS still provides a very short duration snapshot of vehicle exhaust concentrations and is therefore an inherently noisy measurement on an individual vehicle basis, regardless of the accuracy of the instruments used. In contrast, the plume regression approach has a much longer sampling duration of ≈20 s based on the plume profile shown in Figure 1. However, the “noise” is related more to plume interference. We return to this aspect of plume regression and RS in the section *Comparison with Remote Sensing* where the emission uncertainties of each approach are considered. Similar to traditional remote sensing, a single measurement of a vehicle using the plume regression approach is insufficient to characterize its emissions.

### Data Processing

To account for the different response times of the HT8700E and the ICAD, the 10 Hz NH_3_ signal was convoluted using the base R convolve function, prior to the regression analysis. This approach generated NH_3_ plume profiles with a similar shape to the NO_*x*_ and CO_2_ plume profiles. Further details can be found in the Supporting Information.

Robust linear regression was performed in R using the *MASS* package.^23^ The NH_3_, NO_*x*_ and CO_2_ regression coefficients for each vehicle category were used to determine average molar NH_3_/CO_2_ and NO_*x*_/CO_2_ emission ratios. Fuel-specific NH_3_ and NO_*x*_ EFs, expressed in grams of pollutant per kilogram of fuel consumed, were derived from the molar ratios by assuming CO_2_ EFs of 3.16 and 3.17 kg CO_2_ per kg of fuel for diesel and gasoline vehicles, respectively.^24^

For the concentration source apportionment analysis, per vehicle contributions of each vehicle group to roadside NO_2_ increments were determined by multiplying the regression coefficients by the plume weightings for each class of vehicle considered.

## Results and Discussion

### Road Vehicle Emission Factors

The PS instrumentation was deployed at three locations in York (UK) and over 11,000 vehicle passes were recorded. The data was processed using the plume regression approach and the regression coefficients were used to generate the NH_3_ and NO_*x*_ EFs shown in Table 1. The regression model outputs are provided in Tables S2–S4. Unlike previous PS studies, none of the data was discarded, which maximized the sample sizes (n) shown in Table 1. It should be noted that distance-based emission factors (such as g km^–1^) can be estimated from fuel-based emission factors, which enables comparison against vehicle emission limits as described in Davison et al. (2020).^25^

**Table 1:** Mean NH_3_ and NO_*x*_ Fuel-specific EFs Grouped by Vehicle Type, Fuel Type and Euro Class^a^

Vehicle	Fuel type	Euro class	NH_3_ (g kg^–1^)	NO_*x*_ (g kg^–1^)	*n*
bus	diesel	VI	0.51 ± 0.02	0.56 ± 0.11	118
car	diesel	<4	0.02 ± 0.03	12.9 ± 1.45	112
car	diesel	4	0.04 ± 0.03	11.5 ± 1.15	312
car	diesel	5	0.03 ± 0.01	10.6 ± 0.47	934
car	diesel	6	0.04 ± 0.01	7.83 ± 0.43	1137
car	diesel	6*	0.01 ± 0.02	4.01 ± 0.51	214
car	gasoline	<4	0.58 ± 0.07	3.40 ± 0.54	288
car	gasoline	4	0.90 ± 0.09	3.72 ± 0.54	510
car	gasoline	5	0.55 ± 0.04	2.03 ± 0.28	1048
car	gasoline	6	0.52 ± 0.02	1.35 ± 0.16	1578
car	gasoline	6*	0.28 ± 0.02	1.35 ± 0.21	836
hybrid car	gasoline	6	0.33 ± 0.05	1.47 ± 0.40	741
HGV	diesel	<VI	0.00 ± 0.03	21.5 ± 2.87	45
HGV	diesel	VI	0.05 ± 0.02	10.5 ± 0.81	107
LGV	diesel	4	0.00 ± 0.05	16.5 ± 3.32	124
LGV	diesel	5	0.00 ± 0.03	19.0 ± 2.12	381
LGV	diesel	6	0.06 ± 0.02	8.12 ± 0.64	515
LGV	diesel	6*	0.01 ± 0.02	3.96 ± 0.48	475
motorcycle	gasoline	all	1.24 ± 0.67	4.80 ± 3.80	78

a The 95% confidence intervals in the mean are shown. * refers to vehicles which have undertaken RDE testing and ‘*n*’ denotes the sample size

The vehicle categories in Table 1 are classified by vehicle type, fuel type, Euro standard and Real Driving Emissions (RDE) test status. Road vehicle emissions have been regulated in the UK according to European Type Approval emission standards (Euro standards) since 1992.^26^ Euro standards extend from Euro 1/I to Euro 6/VI and have increasingly stringent emission limits and testing methods,^27^ as summarized in Figure 1 of a study by Wilson et al.^28^ Earlier Euro standards were enforced during Type Approval testing under standardized laboratory conditions but a major change occurred in 2017 with the implementation of RDE testing; this relies on the use of a Portable Emissions Measurement System to check the emissions behavior of vehicles under real driving conditions.^29,30^

A benefit of the regression model is that it outputs standard error estimates and *p*-values. The standard errors were multiplied by 1.96 and converted into g kg^–1^ units to give estimates of the 95% confidence intervals in the mean, as shown in Table 1. Even for highly disaggregated data, where sample sizes consist of a few hundred vehicle measurements, the 95% confidence intervals are sufficiently small to distinguish emissions behavior of different vehicle groups, such as Euro 4 and Euro 5 gasoline cars. A comparison of the uncertainties associated with the new plume regression approach and traditional RS measurements is discussed further in the next section.

The p-values provide insight into the statistical significance of the regression coefficients for CO_2_, NO_*x*_ and NH_3_ and low *p*-values indicate that the null hypothesis (no statistical significance between the concentration increments for different vehicle groups) can be rejected. The *p*-values associated with the CO_2_ and NO_*x*_ regression coefficients were highly significant (<7.0 × 10^–7^) for all vehicle groups. For NH_3_, some vehicle groups were associated with *p*-values which exceeded the statistically highly significant threshold of *p* < 0.001, including pre-Euro 4 diesel cars (0.17), Euro 4 diesel cars (0.002), Euro 6 RDE diesel cars (0.30), Euro 4 diesel LGVs (0.008) and Euro 6 diesel LGVs (0.67). *P*-values for the remaining vehicle groups were below 1.7 × 10^–6^. The higher *p*-values for the NH_3_ coefficients are associated with diesel vehicles that have negligible NH_3_ emissions and smaller sample sizes, therefore differences in emissions between these groups are harder to quantify. Slightly negative NH_3_ EFs were determined for pre-Euro VI HGVs and Euro 5 and 6 LGVs; this arises as the NH_3_ emission from the vehicle of interest is zero (or close to zero) and the turbulence generated by the vehicle acts to dilute the NH_3_ emissions to a concentration slightly below the predicted background level. The NH_3_ EFs for these vehicles have been set to 0 g kg^–1^ in Table 1.

NH_3_ emissions were found to be most significant for gasoline vehicles (0.3–0.9 g kg^–1^) and in agreement with previous studies. For example, Farren et al. determined NH_3_ EFs of 0.5–0.8 for Euro 4–6 gasoline cars using traditional RS measurements collected at 37 UK locations between 2017 and 2020,^31^ while RS measurements conducted in China in 2017 reported NH_3_ EFs from gasoline cars ranging from 0.3–0.5 g kg^–1^ for China 0–V emission standards.^32^ NO_*x*_ emissions were higher than NH_3_ emissions, especially for diesel vehicles, yet both pollutants show a clear reduction with Euro standard for a particular vehicle type. The plume regression approach is powerful enough to detect subtle changes in emission behavior associated with developments in emissions legislation, even when sample sizes are modest. For example, emissions from pre- and post-RDE Euro 6 vehicles can be clearly distinguished (0.52 g kg^–1^ vs 0.28 g kg^–1^ of NH_3_ for gasoline cars and 7.83 g kg^–1^ vs 4.01 g kg^–1^ of NO_*x*_ for diesel cars).

Vehicular NO_*x*_ and NH_3_ are important anthropogenic sources of reactive nitrogen (Nr). The contribution of NH_3_ to Nr is more important for gasoline cars than diesel cars. For example, there is 0.027 mol N per kg fuel from Euro 6 RDE diesel car NO_*x*_ and NH_3_ emissions on average, 1.8% of which comes from NH_3_. Conversely, Euro 6 RDE gasoline cars emit 0.022 mol N per kg fuel (from NO_*x*_ and NH_3_), 60.2% of which is attributed to NH_3_. For Euro 6 pre-RDE gasoline cars, the Nr contribution from NH_3_ (0.025 mol N per kg fuel) is 2.8 times higher than from NO_*x*_ (0.009 mol N per kg fuel). In 2015, Bishop and Stedman studied Nr trends in three US fleets and found that Nr emissions from newer model year vehicles were dominated by NH_3_.^33^ This was due to a modest reduction in NH_3_ emissions compared to NO_*x*_ for new gasoline vehicles and increased NH_3_ emissions from aging vehicles with active catalytic converters. Since the Dieselgate scandal, there has been significant growth in the share of new gasoline car registrations at the expense of diesel in the UK,^34^ which will further increase the importance of the contribution of vehicular NH_3_ to the Nr budget.

Further disaggregation of vehicle groups would be possible, e.g., by vehicle manufacturer, model year and engine size, by applying the plume regression approach to a larger PS data set. This is highly feasible given the straightforward deployment of the PS instrumentation and the fact that data associated with overlapping plumes does not need to be discarded.

### Comparison with Remote Sensing

**Figure 3:**
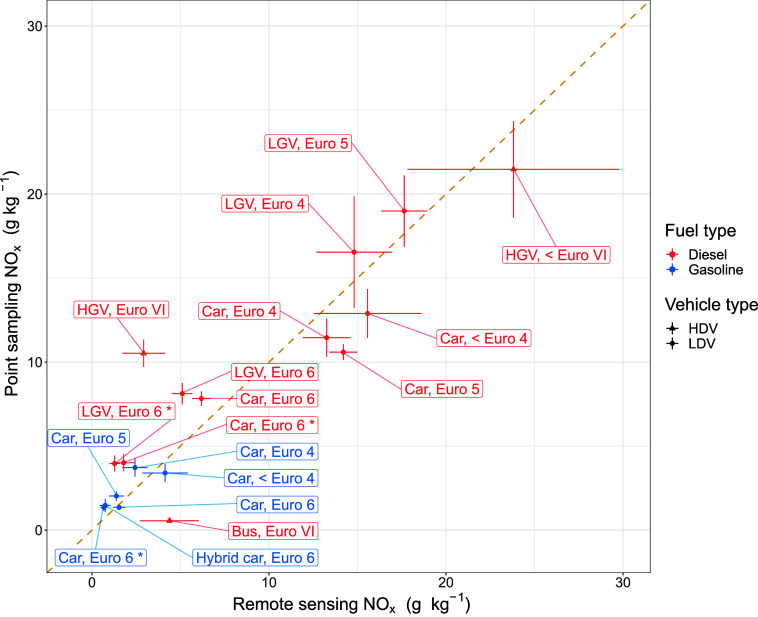
Comparison of mean fuel-specific NO_*x*_ emission factors (g kg^–1^ fuel) from RS and PS measurements. The error bars represent the 95% confidence intervals in the mean.

The PS NO_*x*_ EFs and their associated uncertainties were compared with NO_*x*_ EFs and uncertainties previously established using traditional RS. Figure 3 shows mean fuel-specific NO_*x*_ EFs obtained from RS and PS measurements for a vast range of vehicle groups. The RS EFs were extracted from a data set containing over 170,000 RS measurements of individual vehicles recorded at 23 measurement sites across the UK since 2020. To ensure a fair comparison of uncertainties, subsamples of RS measurements were randomly chosen for each vehicle category, such that the number of measurements selected corresponded to the PS sample sizes (*n*) in Table 1.

Figure 3 shows good agreement between the PS and RS NO_*x*_ EFs across a broad range of vehicle groups, as shown by the close proximity of the data points to the 1:1 reference line. Despite the modest sample sizes, this trend is consistent across high emitting vehicles such as pre Euro VI HGVs and low emitting vehicles such as Euro 6 cars. It should be noted that exact agreement between RS and PS EFs is not expected, due to inherent differences in the data sets (different instrumentation, driving conditions, measurement locations and so on). The average NO_*x*_ EFs for the diesel vehicle groups were 10.1 and 10.6 g kg^–1^ for the RS and PS measurements respectively, while for gasoline vehicles the average NO_*x*_ EFs were 1.82 g kg^–1^ for RS and 2.22 g kg^–1^ for PS. These results provide strong evidence that the plume regression approach is highly robust and can be suitably applied to a vehicle fleet containing a mixture of low, medium and high emitting vehicles.

There are some vehicle groups which deviated further from the 1:1 line, including diesel Euro VI buses and diesel Euro VI HGVs. Further investigation of the associated vehicle technical information and driving conditions revealed potential reasons for these differences. The average NO_*x*_ EFs for the Euro VI buses were 4.38 g kg^–1^ for RS and 0.56 g kg^–1^ for PS. The median manufacture dates were September 2017 and October 2022 for RS and PS respectively. This suggests that the buses measured by PS contained a high fraction of Euro VI stage E buses, which was not the case for the buses measured by RS. Euro VI stage E buses have improved regulatory provisions aimed at reducing NO_*x*_ emission,^35^ however may be prone to higher NH_3_ emissions, which is discussed in more detail later in this study.

For diesel Euro VI HGVs, the average NO_*x*_ EFs determined by RS and PS were 2.92 and 10.5 g kg^–1^ respectively. We speculate two likely reasons for this difference. First, the mean ambient temperature for the RS HGV measurements was 13.4 °C, whereas the mean ambient temperature for the PS HGV measurements at Clifton Moor Gate (where 88% of total HGVs were measured) was 7.8 °C. Therefore, it is possible that the higher NO_*x*_ emissions for the PS HGVs were due to a low temperature NO_*x*_ penalty.^36^ Second, there was a junction from an industrial estate located just before Clifton Moor Gate, and a significant proportion of passing HGVs exited this junction prior to their measurement. It is possible that the aftertreatment systems on these HGVs had not reached their optimum operating temperature. The median speed of the Euro VI diesel HGVs measured by PS was 27.8 km h^–1^. HGVs traveling below the median speed were more likely to have exited the junction and have a cold engine. NO_*x*_ EFs for Euro VI diesel HGVs traveling above and below the median speed were 5.3 and 14.6 g kg^–1^ respectively. Therefore, we speculate that the higher NO_*x*_ emissions for the PS HGVs may also have been caused by a higher proportion of cold start emission measurements.

RS measurements are inherently noisy on an individual vehicle basis due to their short duration. On the other hand, PS measurements are longer (typically a plume is measured for around 20 s), but there is potentially more uncertainty due to interfering plumes from vehicles driving past shortly after one another. The 95% confidence intervals in the mean for RS and PS NO_*x*_ EFs are shown by the error bars in Figure 3. Overall the PS EF uncertainties are smaller than the RS EF uncertainties. The average absolute uncertainties for the vehicle groups shown in Figure 3 are 1.27 and 0.92 g kg^–1^ for RS and PS respectively, which equates to relative uncertainties of 23.4% and 13.1%. This trend remains consistent across the two fuel types; average relative uncertainties for diesel vehicles are 20.0% and 11.4% for RS and PS respectively and for gasoline vehicles, average relative uncertainties are 30.3% for RS and 16.4% for PS. The lower uncertainties associated with the new plume regression approach are a highly useful attribute, as techniques that can more accurately quantifying lower-emitting vehicles are increasingly sought after. It should be noted that the regression-based uncertainties do not account for autocorrelation, which would tend to increase uncertainty.

### Relationship between NH_3_ and NO_*x*_ Emissions

**Figure 4:**
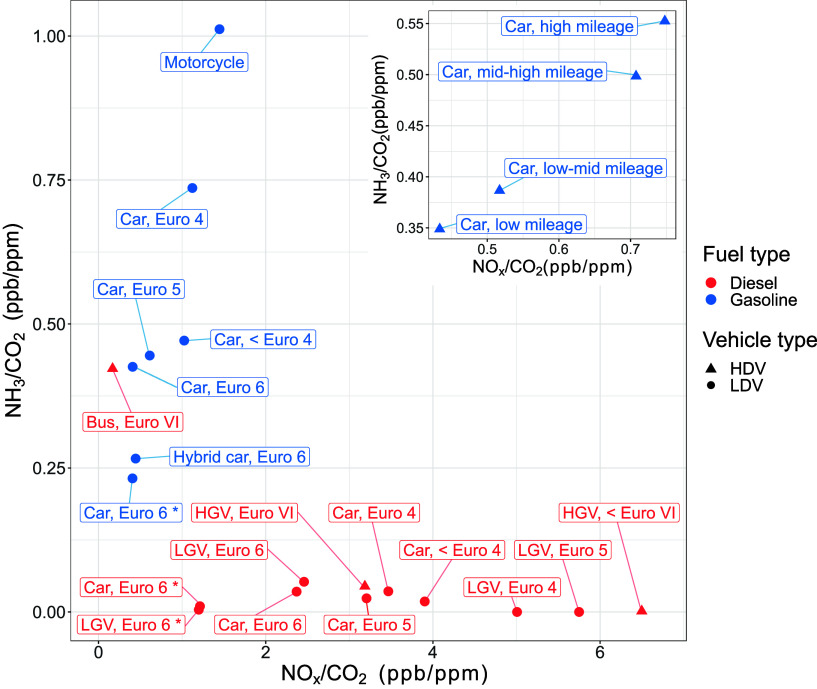
Mean NO_*x*_/CO_2_ and NH_3_/CO_2_ emission ratios determined using the plume regression approach. Vehicles are grouped by vehicle type, fuel type and Euro standard. * refers to vehicles which have undertaken RDE testing. The inset plot shows NO_*x*_/CO_2_ vs NH_3_/CO_2_ emission ratios for gasoline cars by mileage group, derived by merging Euro 4 to 6 data and splitting into mileage quartiles.

Figure 4 shows the mean NO_*x*_/CO_2_ vs NH_3_/CO_2_ emission ratios determined using the plume regression approach, grouped by vehicle type, fuel type and Euro standard, and reveals several important findings. First, a clear distinction between diesel and gasoline vehicles is observed. With the exception of diesel Euro VI buses, diesel vehicles tend to have much lower NH_3_ emissions and higher NO_*x*_ emissions than gasoline vehicles, which are associated with low NO_*x*_ emissions and elevated NH_3_ emissions. This indicates that there is a “trade-off” between NO_*x*_ and NH_3_ emissions and it is challenging to fully suppress emissions of both pollutants for a particular vehicle type.

Second, NH_3_ and NO_*x*_ emissions both increase with increasing vehicle mileage for gasoline cars. The inset plot of Figure 4 shows another example of how the PS data can be grouped into different vehicle categories and contains four vehicle mileage groups for gasoline cars. The deterioration in emissions with vehicle mileage is clear. Each mileage group represents 25% of the observations; the emission ratios associated with the “low mileage” group are derived from gasoline cars with the lowest 25% of miles driven, and so on. The increases in NH_3_/CO_2_ and NO_*x*_/CO_2_ ratios from the lowest to the highest mileage group were 0.20 ppb ppm^–1^ and 0.31 ppb ppm^–1^ respectively. Although the observed rates of NH_3_ and NO_*x*_ deterioration are different, they have a linear relationship and stay proportional to each other across the mileage groups (adjusted *R*^2^ = 0.97, *p*-value <0.01).

Increases in vehicle exhaust emissions as a function of mileage can generally be linked to the deterioration of emission control systems and aftertreatment technologies. For gasoline vehicles, engine-out NO_*x*_ emissions are minimized through reduction reactions on a TWC. However, under certain conditions, excess reduction of NO_*x*_ can occur, which means the target product of N_2_ is missed and NH_3_ is formed as an unintended byproduct.^2^ The efficiency of the TWC depends on the ratio of air to fuel and there is a small operating window around the stoichiometric ratio of air to fuel (λ = 1) where TWCs operate most efficiently. Over time, deterioration of components such as the catalyst surface and the lambda sensor can lead to poorer control over the air to fuel ratio; elevated NO_*x*_ emissions are then typically observed during during lean combustion (λ > 1) while elevated NH_3_ emissions are associated with fuel rich conditions (λ < 1).^2,37^

The Handbook of Emission Factors for Road Transport (HBEFA) provides EFs for all current vehicle categories for a variety of traffic situations and is used widely across Europe for national inventory reports and air quality modeling.^38^ The latest version (HBEFA 4.2) contains emission deterioration functions for CO, NO_*x*_ and NO_2_ but a complete update of mileage correction factors is expected to be included in HBEFA 5.1 (due for publication in mid-2025). It is important that NH_3_ deterioration functions are closely scrutinized prior to this, especially considering the shift to gasoline since the Dieselgate scandal (6.5% of cars registered for the first time in the UK in 2023 were diesel compared to 48.3% in 2015).^34^

Third, Figure 4 shows that NO_*x*_ emissions from diesel vehicles generally decrease with increasing Euro class and NH_3_ emissions remain low, but Euro VI buses are an exception to this trend. Modern diesel vehicles are equipped with SCR systems, which use an urea-based solution to reduce NO_*x*_. However, the injection of urea must be carefully managed to avoid “ammonia slip”, whereby unreacted NH_3_ from the urea is emitted from the tailpipe. In this study, the majority of SCR-equipped diesel vehicles have negligible NH_3_ emissions even when NO_*x*_ emissions are low (such as Euro 6 cars and LGVs) but the Euro VI buses have a high NH_3_/CO_2_ ratio of 0.42 ppb/ppm. Based on the vehicle manufacturer dates, a high proportion of the measured buses are expected to be Euro VI stage E buses. Compared to Euro VI stage D, these have improved regulatory provisions aimed at reducing NO_*x*_ emissions during cold start situations and at lower engine load.^35^ However, this involves injecting the urea-based solution into the SCR at lower temperatures, which may lead to more frequent occurrences of NH_3_ slip, especially under urban driving conditions, which have a higher proportion of stop-start, low speed and engine idling events.

### Concentration Source Apportionment

Aside from the quantification of road vehicle emission factors, an important benefit of PS over RS is that it provides a route to concentration source apportionment. As PS measurements produce a high resolution time series of measured pollutant concentrations at the roadside, the increment above local background, also known as the roadside increment, can be determined for each pollutant. The roadside increment essentially provides an estimate of the concentration of a pollutant that can be attributed to vehicles in the local vicinity, i.e., the traffic driving directly past the PS instrumentation. An additional step made possible by the plume regression approach is to apportion the roadside increment to particular vehicle types, using the coefficients (represented by *a*, *b* and *c* in eqs 1–3) output by the regression model.

**Figure 5:**
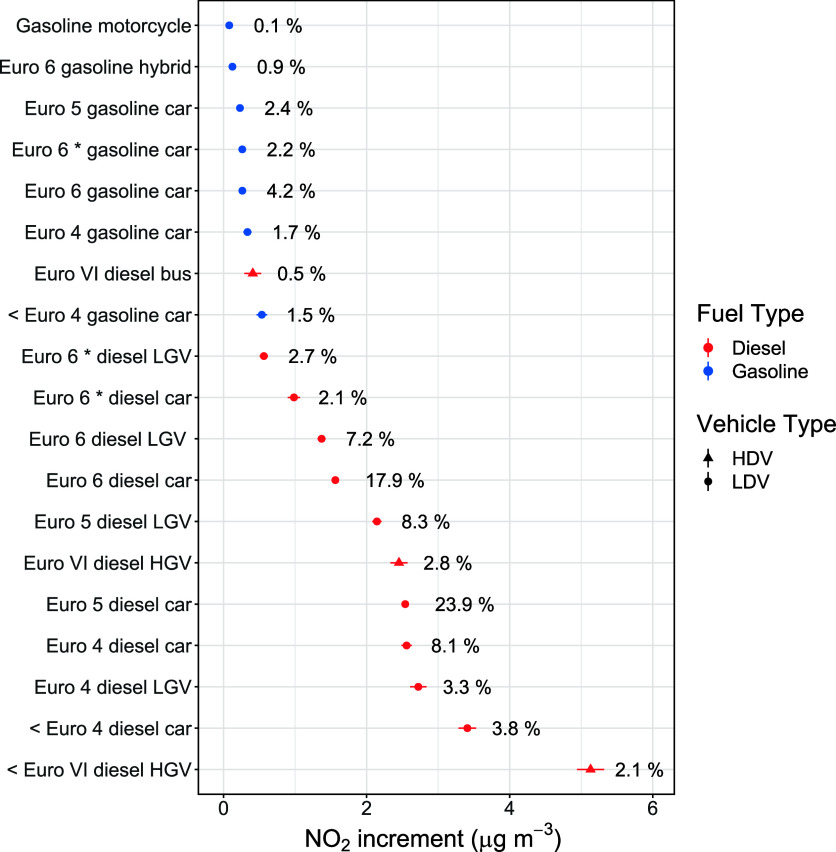
Mean per-vehicle roadside NO_2_ increment concentrations for different vehicle groups, determined using the plume regression approach. The percentages show the contribution of each vehicle type to the total roadside NO_2_ increment. * refers to vehicles which have undertaken RDE testing.

Here we consider source apportionment of the roadside increment of NO_2_ (measured directly by the ICAD at the roadside). Due to the harmful effects of NO_2_ on human health, many countries (including the UK) set air quality objectives for NO_2_ to aim to reduce the amount to which we are exposed.^39^ The median roadside increment of NO_2_ for the PS measurements was 1.4 ppbv, or 2.7 μg m^–3^. Figure 5 shows the average roadside NO_2_ increments on a per vehicle basis for different vehicle categories. The vehicle categories are chosen when the model is constructed and the NO_2_ increments for each category are generated using concentration data that is collected *only* when these vehicles pass the PS instrumentation (≈25 s of data for each vehicle pass, as demonstrated in Figure 1).

Figure 5 shows that on a per vehicle basis, pre Euro VI diesel HGVs contribute to the highest amount of NO_2_ exposure at the roadside. Other significant contributors include Euro 4 diesel LGVs and pre Euro 4 diesel cars. There is a clear distinction between the contribution of diesel and gasoline vehicles; many gasoline vehicles such Euro 5 and 6 cars contribute significantly less to roadside NO_2_ exposure on a per vehicle basis.

An important consideration is vehicle fleet composition. The per-vehicle NO_2_ increments were scaled by the fleet composition to calculate the percentage contribution of each vehicle type to the total roadside NO_2_ increment, as shown by the percentages next to each data point in Figure 5. The percentages will vary according to the measured vehicle fleet composition at a given location. Although pre Euro VI diesel HGVs contributed most to NO_2_ increments on a per vehicle basis, they made up just 0.4% of the fleet in this study and therefore had a low contribution to the total vehicular NO_2_ increment (2.1%). Euro 5 and Euro 6 diesel cars comprised 9.2% and 11.2% of the measured fleet respectively, and were found to be the dominant contributors to the total NO_2_ increment (23.9% for Euro 5 diesel cars and 17.9% for Euro 6 diesel cars). The percentage contribution to roadside NO_2_ increment for vehicle types such as Euro 6 gasoline hybrids and Euro 6 RDE gasoline cars remained low (≈1–2%) despite their significant contribution to the fleet composition (7.3% and 8.3% respectively).

### Recommendations and Outlook

Looking ahead, the plume regression approach could be widely adopted around the world, through the deployment of fast response analyzers measuring a wide range of air pollutants and CO_2_ at roadside monitoring sites. This approach offers a potentially cost-effective solution for cities to upgrade their air quality monitoring stations to better understand transport emissions and develop data-driven policies. The existing capability of roadside monitoring sites to provide hourly air pollution concentrations for comparison with air quality health based thresholds would significantly be enhanced by also providing vehicle emissions quantification and determining how much different vehicles are contributing to ambient air pollution concentrations. It is recommended that a program of renewing roadside air pollution monitoring considers deploying fast-response analyzers e.g., ICAD but paired with an ANPR camera for vehicle fleet apportionment.

Measurements could be conducted in a pollution hotspot or area proposed to be a low emission zone, and roadside pollutant increments scaled by the location-specific vehicle fleet composition to establish which vehicle types to minimize or exclude to have the most beneficial effect on ambient exposure to harmful pollutants. Direct correlation between fuel-specific EFs and exposure concentrations for given vehicle types is not anticipated, due to complexities associated with vehicle-induced turbulence and plume dilution. Therefore, it is critical that new techniques, such as the point sampling and plume regression approach presented in this study, are developed in order to apportion emissions *and* exposure concentrations of a broad suite of pollutants to a wide range of vehicle types.

## Supporting Information Available

Detail on instrument calibration, site photographs, plume shapes by vehicle type and pollutant, information on regression model coefficients and standard errors, and convolution of the ammonia measurements (PDF)
