# Supplementary material for: An Ambient Measurement Technique for Vehicle Emission Quantification and Concentration Source Apportionment
Source: Environ Sci Technol. 2024 Nov 4;58(45):20091–100. doi: 10.1021/acs.est.4c07907 (PMC11562735; doi:10.1021/acs.est.4c07907)
Supplement: Supplementary file 1 — es4c07907_si_001.pdf [file es4c07907_si_001.pdf]

An ambient measurement technique for vehicle emission quantification and concentration source apportionment

Naomi J. Farren,[†] Sam Wilson,[†] Yoann Bernard,[‡] Marvin D. Shaw,[†,¶] Kaylin Lee,[‡] Mallery Crowe,[‡] and David C. Carslaw[*,†]

† *Wolfson Atmospheric Chemistry Laboratories, University of York, York, YO10 5DD, United Kingdom*

‡ *The International Council on Clean Transportation, Fasanenstr. 85, 10623 Berlin, Germany*

¶ *National Centre for Atmospheric Science, University of York, York, YO10 5DD, United Kingdom*

E-mail: david.carslaw@york.ac.uk

Summary

Number of pages: 15

Number of Figures: 5

Number of Tables: 4

Supporting Information Available

Figures

Figure S1: PS measurement site 1 (University Road southbound, 53.947, −1.047).

Figure S2: PS measurement site 2 (University Road northbound, 53.947, −1.047).

Figure S3: PS measurement site 3 (Clifton Moor Gate, 53.987, −1.103).

Figure S4: Mean plume profiles for CO_2, NO_x and NH_3, grouped by vehicle type. Time on the x-axis is the time since the front of a vehicle is detected. The plume profiles are derived from vehicle passes with at least a 20 s interval before and after other vehicle passes.

Figure S5: The range of convolution functions tested on the raw NH_3 signal. The shape of the convolution function is e^{-xt}, where x ranges from 0.1 to 1.0 and t is the time in milliseconds.

Figure S6: CO_2 and NH_3 time series for a measured vehicle exhaust plume. The raw NH_3 signal and the convoluted NH_3 signal (created using the $e^{-0.4t}$ function) are shown. A 450 ppm offset has been applied to the CO_2 signal for plotting purposes.

Tables

Table S1: Summary of the measured vehicle fleet composition and driving conditions.

Table S2: Robust regression model output for NH_3. The regression coefficients for each vehicle category are shown.

Table S3: Robust regression model output for NO_x. The regression coefficients for each vehicle category are shown.

Table S4: Robust regression model output for CO_2. The regression coefficients for each vehicle category are shown.

Calibration of the HT8700E NH_3 analyser

The open-air cell of the HT8700E analyser was enclosed using a glass cylindrical tube, with 1/4" diameter ports at each end. An NH_3 permeation tube with a known emission rate (560 ng min^{-1}) and varying flows of N_2 gas (1.5, 2, 3 and 4 L min^{-1}) was used to flush the enclosed cell with known concentrations of NH_3 (537, 403, 269 and 201 ppbv). The permeation tube was housed within a temperature controlled permeation oven (40 $^\circ$C).

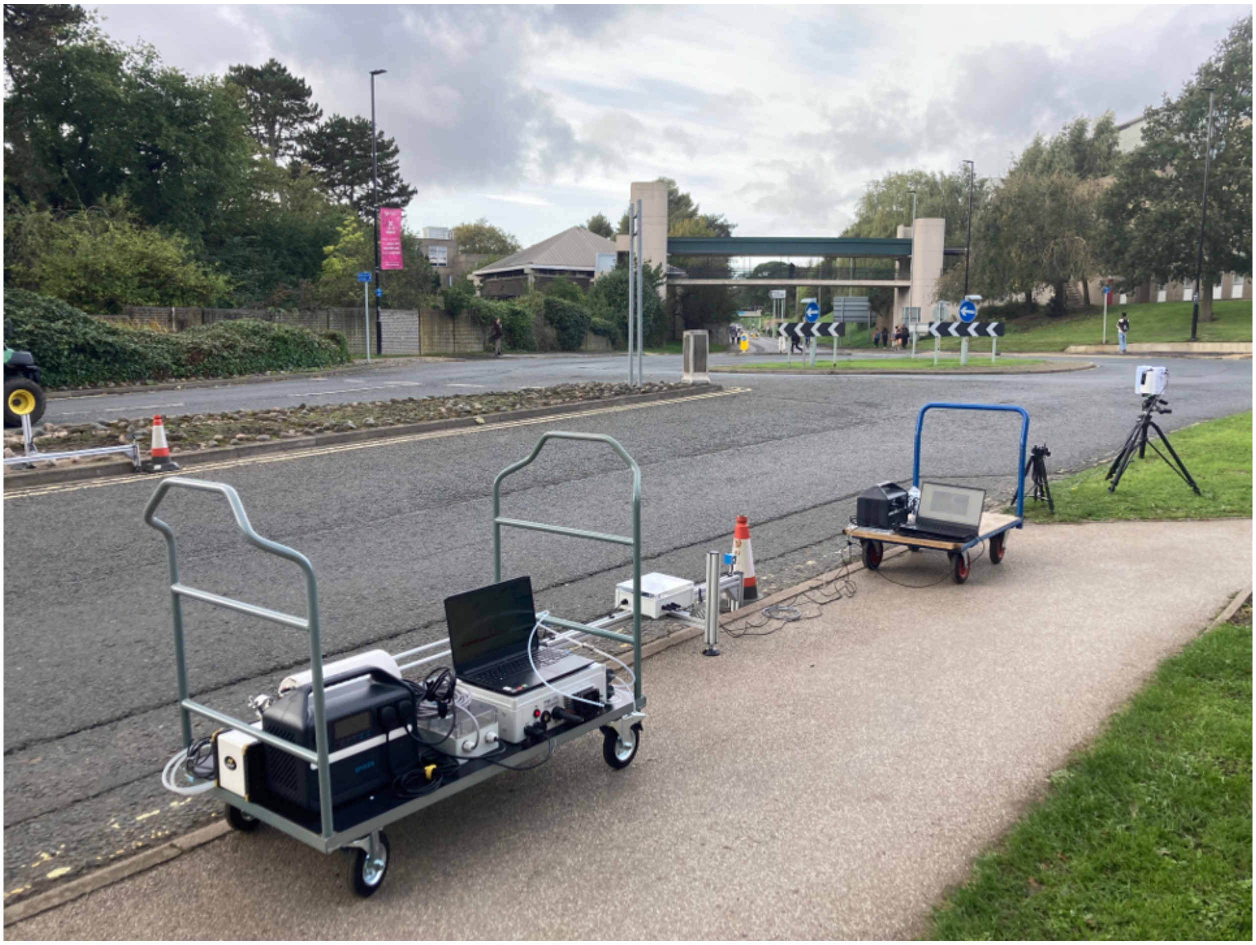

Figure S1: PS measurement site 1 (University Road southbound, 53.947, −1.047).

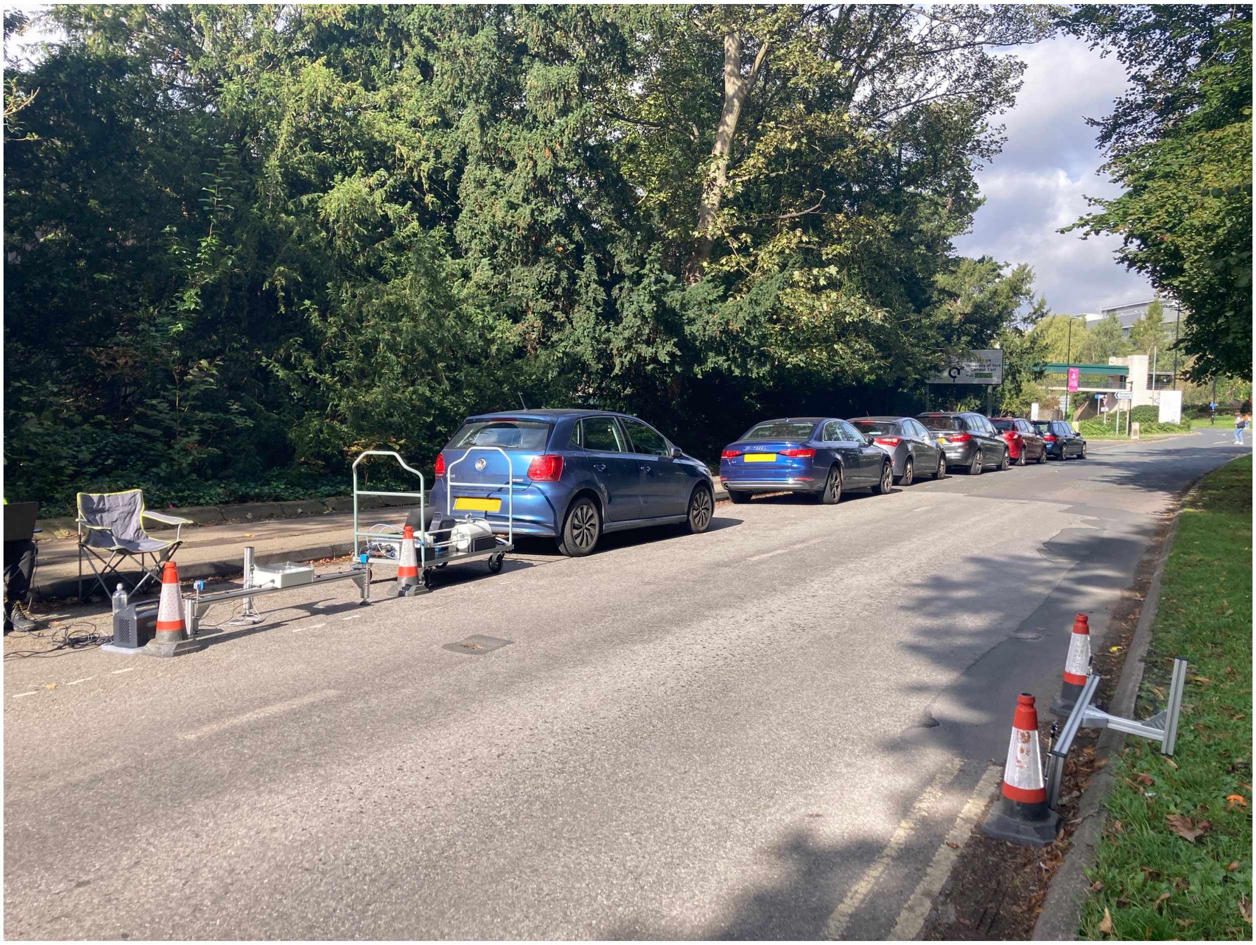

Figure S2: PS measurement site 2 (University Road northbound, 53.947, −1.047.)

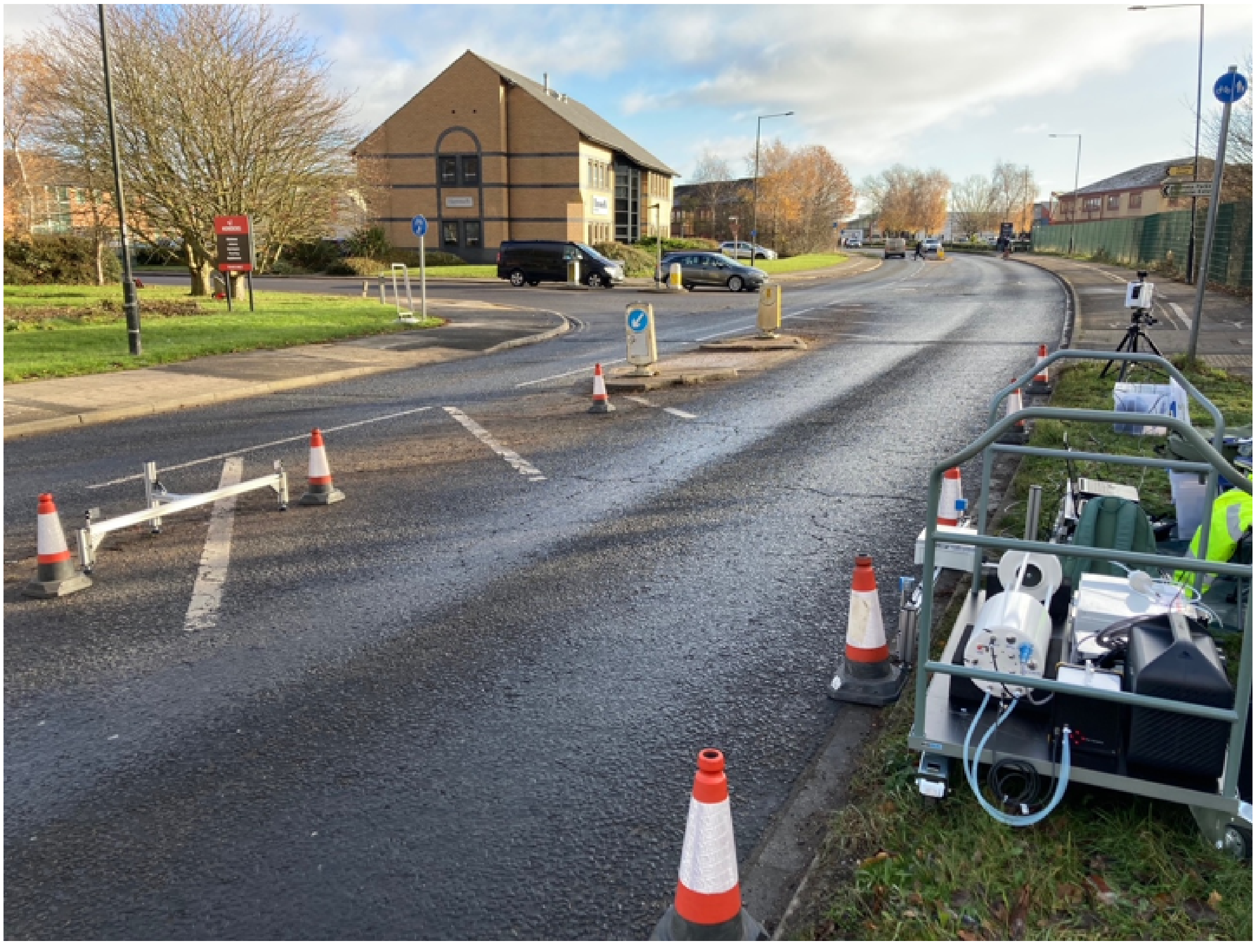

Figure S3: PS measurement site 3 (Clifton Moor Gate, 53.987, −1.103).

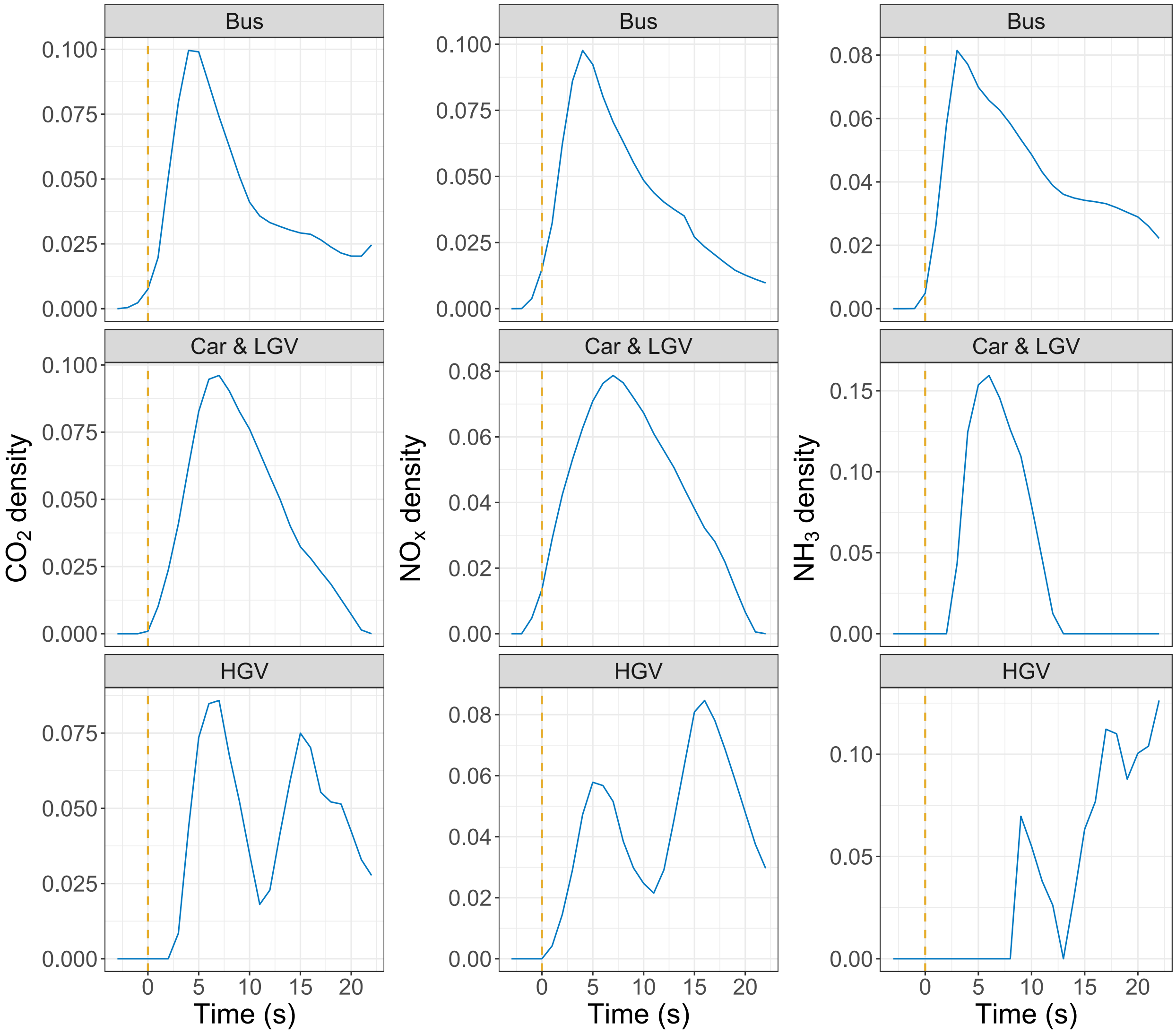

Figure S4: Mean plume profiles for CO_2, NO_x and NH_3, grouped by vehicle type. Time on the x-axis is the time since the front of a vehicle is detected. The plume profiles are derived from vehicle passes with at least a 20 s interval before and after other vehicle passes.

Table S1: Summary of the measured vehicle fleet composition and driving conditions.

**Characteristic**	**Clifton Moor**	**University**
# measurements	6,528	4,107
Diesel (%)	44	42
Gasoline (%)	41	42
Gasoline hybrid (%)	7	9
Electric (%)	3	4
Pre Euro 5 (%)	14	13
Euro 5 (%)	22	26
Euro 6 (%)	59	56
Mean speed ($km\,h^{-1}$)	44.2	34.5
Mean acceleration ($km\,h^{-1}\,s^{-1}$)	4.8	0.9
Mean ambient temperature ($^\circ C$)	7.8	16.3

Table S2: Robust regression model output for NH_3. The regression coefficients for each vehicle category are shown.

Vehicle category	Coefficient	Standard error	t-value
car_petrol_pre_euro_4	19.55	0.45	43.54
car_petrol_euro_4	24.10	0.34	70.38
car_petrol_euro_5	15.08	0.23	65.20
car_petrol_euro_6_pre_rde	18.44	0.19	97.94
car_petrol_euro_6_rde	10.54	0.26	40.49
car_petrol_low_mileage	13.38	0.25	53.95
car_petrol_low_mid_mileage	16.90	0.25	67.38
car_petrol_mid_high_mileage	19.59	0.25	78.94
car_petrol_high_mileage	22.86	0.25	92.09
car_diesel_pre_euro_4	1.02	0.74	1.39
car_diesel_euro_4	1.39	0.44	3.16
car_diesel_euro_5	1.20	0.25	4.81
car_diesel_euro_6_pre_rde	1.43	0.22	6.39
car_diesel_euro_6_rd	0.54	0.52	1.05
car_hybrid_pre_euro_6	4.02	0.75	5.38
car_hybrid_euro_6	7.10	0.28	25.23
lgv_diesel_pre_euro_4	3.39	1.15	2.94
lgv_diesel_euro_4	-1.84	0.69	-2.68
lgv_diesel_euro_5	-1.89	0.39	-4.85
lgv_diesel_euro_6_pre_rde	2.21	0.34	6.53
lgv_diesel_euro_6_rde	0.15	0.35	0.44
hgv_diesel_pre_euro_6	-5.73	1.14	-5.03
hgv_diesel_euro_6	3.62	0.72	5.01
bus_diesel_pre_euro_6	-1.57	1.87	-0.84
bus_diesel_euro_6	81.14	0.71	114.68
motorcycle_petrol	13.68	0.87	15.64

Table S3: Robust regression model output for NO_x. The regression coefficients for each vehicle category are shown.

**Vehicle category**	**Coefficient**	**Standard error**	**t-value**
car_petrol_pre_euro_4	42.52	1.93	21.98
car_petrol_euro_4	36.75	1.48	24.91
car_petrol_euro_5	20.74	1.00	20.82
car_petrol_euro_6_pre_rde	17.69	0.81	21.80
car_petrol_euro_6_rde	18.47	1.12	16.47
car_petrol_low_mileage	16.62	1.07	15.53
car_petrol_low_mid_mileage	22.60	1.08	20.88
car_petrol_mid_high_mileage	27.79	1.07	25.96
car_petrol_high_mileage	30.94	1.07	28.88
car_diesel_pre_euro_4	218.91	3.17	69.04
car_diesel_euro_4	134.25	1.89	71.13
car_diesel_euro_5	162.48	1.08	151.09
car_diesel_euro_6_pre_rde	96.06	0.97	99.52
car_diesel_euro_6_rde	66.19	2.23	29.69
car_hybrid_pre_euro_6	-23.11	3.22	-7.17
car_hybrid_euro_6	11.83	1.21	9.76
lgv_diesel_pre_euro_4	318.46	4.97	64.07
lgv_diesel_euro_4	139.43	2.97	47.02
lgv_diesel_euro_5	158.87	1.68	94.46
lgv_diesel_euro_6_pre_rde	103.68	1.46	71.15
lgv_diesel_euro_6_rde	47.43	1.53	31.07
hgv_diesel_pre_euro_6	427.72	4.91	87.17
hgv_diesel_euro_6	270.42	3.12	86.78
bus_diesel_pre_euro_6	402.28	8.07	49.83
bus_diesel_euro_6	32.55	3.05	10.68
motorcycle_petrol	19.57	3.77	5.19

Table S4: Robust regression model output for CO_2. The regression coefficients for each vehicle category are shown.

Vehicle category	Coefficient	Standard error	t-value
car_petrol_pre_euro_4	41.48	1.47	28.19
car_petrol_euro_4	32.74	1.12	29.17
car_petrol_euro_5	33.86	0.76	44.67
car_petrol_euro_6_pre_rde	43.32	0.62	70.20
car_petrol_euro_6_rde	45.43	0.85	53.26
car_petrol_low_mileage	38.32	0.81	47.05
car_petrol_low_mid_mileage	43.70	0.82	53.05
car_petrol_mid_high_mileage	39.27	0.81	48.20
car_petrol_high_mileage	41.38	0.82	50.74
car_diesel_pre_euro_4	56.10	2.41	23.25
car_diesel_euro_4	38.73	1.44	26.97
car_diesel_euro_5	50.68	0.82	61.94
car_diesel_euro_6_pre_rde	40.54	0.73	55.21
car_diesel_euro_6_rde	54.55	1.70	32.16
car_hybrid_pre_euro_6	14.18	2.45	5.78
car_hybrid_euro_6	26.65	0.92	28.90
lgv_diesel_pre_euro_4	70.72	3.78	18.70
lgv_diesel_euro_4	27.85	2.26	12.34
lgv_diesel_euro_5	27.64	1.28	21.60
lgv_diesel_euro_6_pre_rde	42.17	1.11	38.03
lgv_diesel_euro_6_rde	39.54	1.16	34.04
hgv_diesel_pre_euro_6	65.82	3.73	17.63
hgv_diesel_euro_6	84.88	2.37	35.80
bus_diesel_pre_euro_6	26.58	6.14	4.33
bus_diesel_euro_6	193.76	2.32	83.54
motorcycle_petrol	13.52	2.87	4.72

Ammonia signal convolution

The signal rise and fall times of the HT8700E NH_3 analyser are quicker than those of the ICAD NO_x and CO_2 analyser. This is expected because the HT8700E is an open-path analyser which provides real-time data at 10 Hz resolution, whereas the ICAD is a closed-path analyser with a response time (10% to 90%) of 2 s. It is important to account for the differing analyser response times ahead of the robust regression analysis and generate NH_3 plume profiles which have a similar shape to the NO_x and CO_2 plume profiles. In this study we achieve this by applying a convolution function to the raw NH_3 signal. A similar approach has previously been adopted by Brimblecombe et al.[1]

The most appropriate convolution function was found using a trial and error approach; Figure S5 shows a range of convolution functions that were applied to the raw NH_3 concentration data. The function which generated a convoluted NH_3 signal with the highest correlation to the NO_x and CO_2 signals was selected ($e^{-0.4t}$). Figure S6 shows the measured NH_3 and CO_2 signals and the convoluted NH_3 signal for a typical plume produced by a passing vehicle. The profile of the convoluted NH_3 signal closely follows that of CO_2. The raw NH_3 time series data was convoluted using the base R convolve function prior to the robust regression analysis.

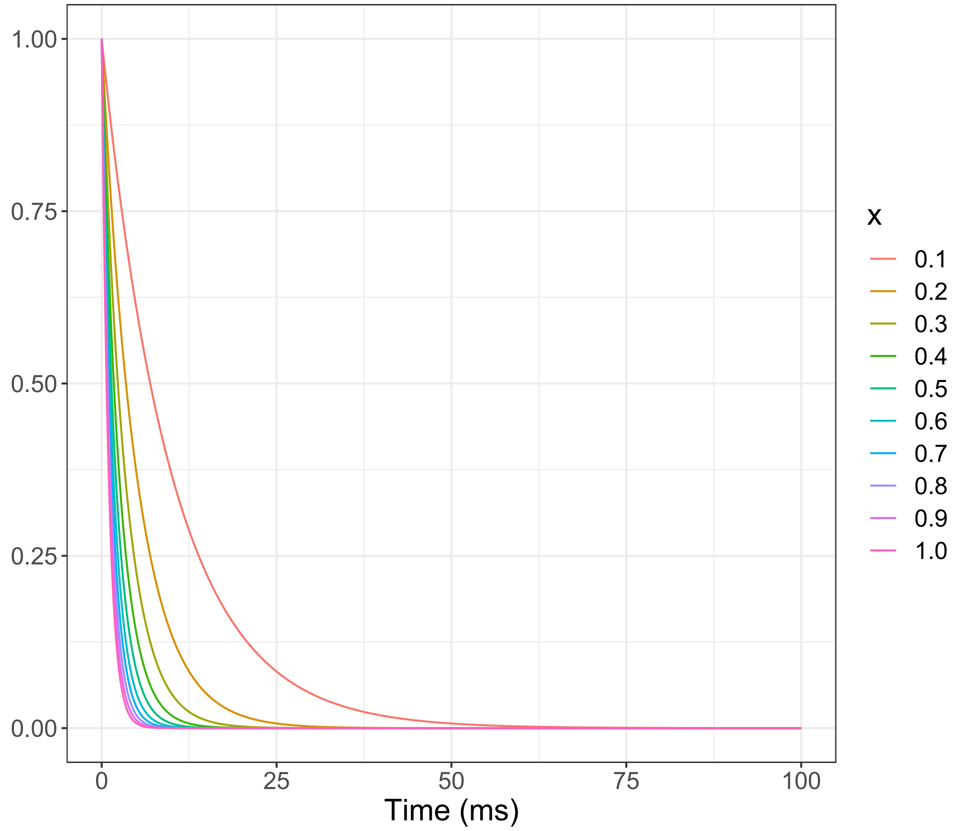

Figure S5: The range of convolution functions tested on the raw NH_3 signal. The shape of the convolution function is e^{-xt}, where x ranges from 0.1 to 1.0 and t is the time in milliseconds.

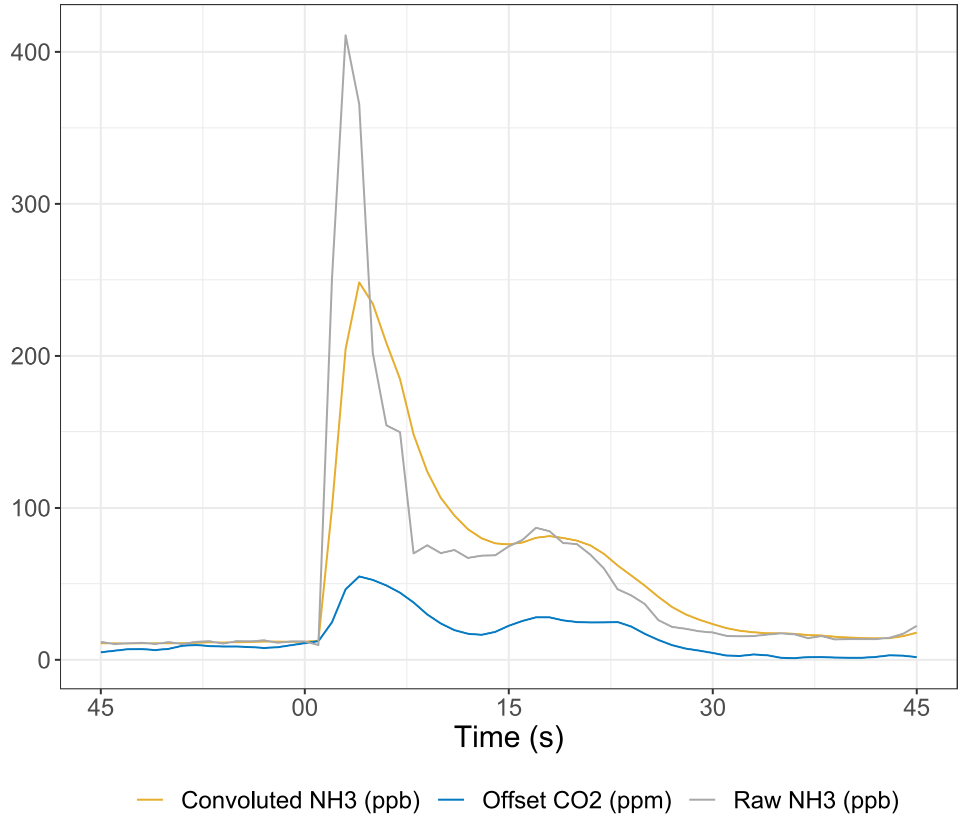

Figure S6: CO_2 and NH_3 time series for a measured vehicle exhaust plume. The raw NH_3 signal and the convoluted NH_3 signal (created using the $e^{-0.4t}$ function) are shown. A 450 ppm offset has been applied to the CO_2 signal for plotting purposes.

References

(1) Brimblecombe, P.; Chu, M.; Liu, C.-H.; Fu, Y.; Wei, P.; Ning, Z. Roadside NO2/NOx and primary NO2 from individual vehicles. *Atmospheric Environment* **2023**, *295*, 119562.
